# Can Primary Care Drive Tuberculosis Elimination? Increasing Latent Tuberculosis Infection Testing and Treatment Initiation at a Community Health Center with a Large Non-U.S.-born Population

**DOI:** 10.1007/s10903-022-01438-1

**Published:** 2023-01-18

**Authors:** Amy S. Tang, Tessa Mochizuki, Zinnia Dong, Jennifer Flood, Shereen S. Katrak

**Affiliations:** 1 North East Medical Services, 1520 Stockton St., San Francisco, CA 94133 USA; 2 Tuberculosis Control Branch, California Department of Public Health, Richmond, CA USA; 3 Division of Infectious Diseases, University of California, San Francisco, San Francisco, CA USA

**Keywords:** Latent tuberculosis infection, Tuberculosis screening, Community health center, Primary care, Non-U.S.-born

## Abstract

Community health centers (CHC) play a key role in latent tuberculosis infection (LTBI) testing and treatment. We performed a retrospective analysis of LTBI testing and treatment among pediatric and adult patients at a CHC with a large non-U.S.-born (USB) population during a series of quality improvement (QI) interventions from 2010 to 2019. Among 124,695 patients with primary care visits, 40% of patients were tested for tuberculosis (TB) infection and among those tested, 20% tested positive, including 39% of adults aged 50–79 years. Compared to adults aged 18–49 years, children aged 6–17 had increased odds of LTBI testing and treatment initiation [odds ratio and 95% confidence interval 3.23 (3.10, 3.36) and 1.41 (1.12, 1.79), respectively], while age ≥ 65 was associated with lower odds of both testing and treatment initiation. Over the analysis period, coinciding with unfunded QI interventions intended to reduce barriers to LTBI care, there was a significant increase in the proportion of patients receiving LTBI testing for both adults (6% to 47%, p < 0.001) and children (23% to 80%, p < 0.001). During the analysis period, there was also a significant increase in the proportion of patients receiving prescriptions for LTBI treatment, as well as provider use of evidence-based strategies including rifamycin-based treatment. Our study suggests that primary care interventions can reduce barriers to LTBI treatment and drive TB elimination.

**Supplementary Information:**

The online version contains supplementary material available at 10.1007/s10903-022-01438-1.

## Background

Until the COVID-19 pandemic in 2020, tuberculosis (TB) was the leading infectious cause of death worldwide despite the availability of effective treatments [1]. In 2019, there were 8916 TB disease cases reported in the United States (U.S.), with California accounting for 2113 (24%) of total cases, more cases than any other state [2]. In California, half of individuals diagnosed with TB are hospitalized and 10% of individuals with TB disease do not survive TB treatment [3, 4]. TB is both a health equity issue and a preventable and costly burden on the healthcare system [5, 6]. Cost effectiveness studies show that TB interventions focused on non-U.S.-born (USB) individuals in California could avert 9800 TB cases and $179 million treatments costs over 29 years [7].

The leading strategy for eliminating TB in the U.S. is to identify and treat persons with latent TB infection (LTBI). Diagnosing and treating LTBI can prevent progression to TB disease and prevent transmission to others [8]. In 2016, the U.S. Preventive Services Task Force updated the LTBI testing recommendations to test all populations at increased risk, including persons born in or former residents of countries with increased LTBI prevalence. This includes all countries outside of the U.S., Canada, Australia, New Zealand, and western or northern Europe [9, 10]. Recent clinical guidelines recommend the use of interferon-gamma release assay (IGRA) for testing non-USB patients rather than TB skin test (TST), and prioritization of 3- or 4-month rifamycin-based therapies for treatment rather than 9 months of isoniazid (INH), due to higher rates of completion and lower rates of hepatotoxicity for rifamycin-based therapies [11–13].

While there is little published data on LTBI testing and treatment practices in primary care health systems or clinics, meta-analysis data suggests that only 30% of people at risk for LTBI are started on treatment, and less than 20% complete appropriate treatment [14]. Prior studies done at community health centers (CHC) showed that adopting TB risk assessments, implementing provider education, and involving care coordination have increased LTBI testing and treatment completion; however, these studies generally took place over 1–2 years, with no follow-up [15–17]. The purpose of this retrospective data analysis was to describe LTBI testing and treatment practices during a series of quality improvement (QI) interventions at an urban CHC serving primarily non-USB Asian patients in the San Francisco Bay Area over a decade from 2010 to 2019.

## Methods

### Study Site

North East Medical Services (NEMS) is a federally funded CHC in the San Francisco Bay Area with 12 clinic sites that serve approximately 66,000 patients each year and a predominantly non-USB Asian population at risk for TB infection and disease. NEMS offers comprehensive primary care services including in-house laboratory, pharmacy, and radiology services with bilingual, language-concordant providers who speak Chinese language dialects such as Cantonese, Mandarin, and Taishanese, as well as Vietnamese, Tagalog, and Burmese. In 2013, the San Francisco Department of Public Health (SFDPH) TB Prevention and Control Program approached NEMS about a high proportion of TB disease cases among patients with a NEMS primary care provider. While there was no external funding or financial incentives for improving LTBI testing and treatment, NEMS recognized TB as a major health disparity affecting its non-USB Asian patient population and began a series of QI initiatives to identify and treat patients with LTBI.

### Study Population

The study population included all patients with at least one adult medicine or pediatric provider visit at NEMS from January 1, 2010, to December 31, 2019, and excluded patients without an assigned primary care provider or with only an Obstetrics/Gynecology visit during the study period. Provider visits included well-child visits, adult annual physical exams, chronic care follow-up, as well as urgent care visits with primary care providers.

### QI Interventions

**Fig. 1:**
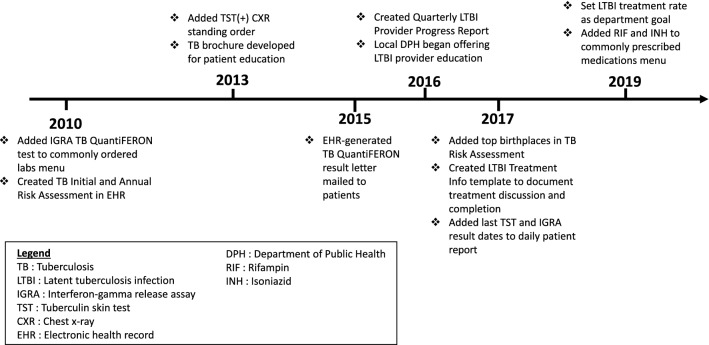
Timeline of LTBI program quality improvement interventions

In 2010, NEMS began implementing a series of clinic-directed interventions, led by a NEMS physician who acted as a “champion” for TB prevention. The first intervention was the addition of an annual TB risk assessment to NEMS’ electronic health record (EHR). The risk assessment included prior TB testing results and chest imaging information and prompted the patient’s medical team to annually screen for TB risk factors such as recent travel to a region with increased TB prevalence, even after baseline testing was performed. The EHR-based annual risk assessment was followed by a series of LTBI QI interventions taken over the next decade (Fig. 1).

EHR-based interventions to reduce barriers to providing LTBI care included updating to the TB Risk Assessment template to focus on the highest TB risk factors, adding the IGRA TB test to the commonly ordered labs menu for ease of provider ordering, generating automatic IGRA result letters mailed to patients to promote timely result notification and activate patients to follow-up on positive or indeterminate results, adding rifampin and INH to the commonly prescribed medications menu to ease provider ordering and standardize administration instructions, and developing a template to document initiation of LTBI treatment and prior TB treatment, and to capture patients’ declination of treatment after risks and benefits discussed. Such changes were discussed at medical provider quality improvement meetings to obtain provider feedback and awareness of TB as a priority issue.

Patients’ last TST or IGRA result and date were added to the daily patient report, which was used to prompt providers and nursing staff to screen for TB if no prior test was performed. Since TST placement and readings were performed by nursing staff, a standing chest X-ray (CXR) order was automatically created for nursing staff to order follow-up chest imaging for positive TST results if there was no CXR on record in the 6 months prior to the positive TST test.

In 2016, NEMS collaborated with the SFDPH TB Prevention and Control Director to provide annual LTBI provider education. Trainings emphasized evidence-based strategies such as use of IGRAs for non-USB patients, and later, use of rifamycin-based therapies. LTBI provider performance measures, such as number of TB tests ordered and percentage follow-up of positive results with CXR and treatment initiation, were added to providers’ quarterly clinical measures progress report. In 2019, the adult medicine department set a target goal for 65% compliance for providers recommending and prescribing LTBI treatment to persons with positive test for TB infection.

NEMS’ health education department also created a TB patient education brochure in 2013 that was distributed to all NEMS clinic sites and exam rooms to supplement discussions with the provider about LTBI testing and treatment.

### Standard LTBI Care Workflow

A standardized LTBI care workflow was created based on guidance from local and state DPH and shared with all primary care providers. Patients with a positive IGRA or TST test result completed a follow-up CXR and TB disease symptom screen. If there was abnormal imaging or the patient had symptoms concerning for TB disease, the patient was referred to the local health department for evaluation and follow-up care. If the follow-up CXR was normal, the NEMS provider offered LTBI treatment and follow-up monitoring until the patient completed treatment. Follow-up monitoring included in-person visits for assessment of treatment adherence and medication-related adverse reactions. The frequency of follow-up visits varied from two visits (at one month after treatment initiation and at treatment completion) to monthly for the duration of treatment, depending on patient’s risk factors for adverse reactions such as hepatotoxicity. Of note, patients with prior positive IGRA or TST with or without treatment were generally not re-tested. Chest imaging was ordered for such patients if they had new risks factors or symptoms.

### Data Collection and Analysis

We retrospectively extracted data on patient demographics and clinical characteristics, clinical encounters, and TB screening, testing, imaging, and treatment from NEMS’ EHR and stored the data on a secure server. We described the demographic and clinical characteristics of patients tested and treated for TB infection, testing and treatment practices by provider and site, and the LTBI care cascade outcomes for 2019. Definitions for each step of the LTBI care cascade can be found in Supplemental Table 1. We assessed temporal trends in LTBI testing, LTBI treatment initiation, and use of preferred practices such as IGRA vs. TST and prescription of rifamycin-based therapies vs. INH over the 10-year analysis period. SAS (version 9.4; SAS Institute, Inc., Cary, North Carolina) was used for descriptive and statistical analysis. Chi-squared test was used for univariate analysis of characteristics associated with testing and treatment. Multivariate logistic regression was used to determine if categorical characteristics were associated with patients being treated. Cochran-Armitage Trend Test was used to assess temporal trends.

Testing for TB infection was defined as the presence of data in the result field for TST or IGRA. TST test interpretation was based on CDC guidelines [18]. TST positivity was defined as an induration of 10 or more millimeters for persons born in a country where TB is common, regardless of years in the U.S. Treatment initiation was defined as an INH or rifamycin prescription in the patient’s chart or completion of a discrete field indicating prior treatment. Non-USB was defined using three EHR fields: place of birth, date of entry to the U.S., and preferred language. If at least one field indicated birth outside the U.S. or date of entry into the U.S., the patient was categorized as non-USB. If the only field available was English as preferred language, non-USB status was categorized as unknown.

### Ethical Approval

The Association of Asian Pacific Community Health Organizations institutional review board reviewed and approved this research.

## Results

There were 124,695 patients with at least one adult medicine or pediatric provider visit at NEMS from 2010 to 2019 (Table 1). Among the study population, 84% identified as Asian and 80% identified as non-USB, with China, Vietnam, Hong Kong, and Philippines representing the top four places of birth. More than two-thirds (68%) of the study population selected Cantonese or Mandarin Chinese as their preferred language. In the adult study population, coexisting health conditions included 8% with hepatitis B virus (HBV), 10% with diabetes mellitus, and 15% as a current smoker. While human immunodeficiency virus (HIV) infection and homelessness are known risk factors for progression to and exposure to TB disease, patients with either risk factor, respectively, comprised less than 1% of this CHC’s patient population and thus were not included in the regression analysis. More than half (57%) of the study population had Medicaid health insurance which included those with dual Medicaid/Medicare; 21% were uninsured and 16% had private insurance.

**Table 1:** Patient demographic and clinical characteristics, testing status for TB infection, and odds of testing among each subgroup 2010–2019

	Overall N (%)*	Tested for TB N (%)*	Percent tested for TB among subgroup	Unadjusted odds ratio (95% CI and p-value)	
Total	124,695	49,767	40		
Gender					
Female	67,706 (54)	28,511 (57)	42	REF	< 0.0001
Male	56,989 (46)	21,256 (43)	37	0.82 (0.80, 0.84)	
Age [median, IQR] (1)	39 [22, 56]	30 [12, 51]			< 0.0001
0–5	11,523 (9)	8433 (17)	73	4.94 (4.72, 5.16)	
6–17	12,384 (10)	7937 (16)	64	3.23 (3.10, 3.36)	
18–49	56,332 (45)	20,060 (40)	36	REF	
50–64	30,347 (24)	10,143 (20)	33	0.91 (0.88, 0.94)	
65–79	11,907 (10)	2853 (6)	24	0.57 (0.54, 0.60)	
≥ 80	2202 (2)	341 (1)	15	0.33 (0.30, 0.37)	
Race and ethnicity					< 0.0001
American Indian/Alaska Native	150 (0.1)	48 (0.1)	32	0.64 (0.45, 0.90)	
Asian	104,352 (84)	44,308 (89)	42	REF	
Black/African American	1770 (1)	455 (1)	26	0.47 (0.42, 0.52)	
Latino	4695 (4)	1574 (3)	34	0.68 (0.64, 0.73)	
Native Hawaiian or Other Pacific Islander	846 (1)	275 (1)	33	0.65 (0.57, 0.75)	
White	7836 (6)	1532 (3)	20	0.33 (0.31, 0.35)	
Unknown	5046 (4)	1575 (3)	31	0.62 (0.58, 0.65)	
Preferred language					< 0.0001
Cantonese	62,588 (50)	30,558 (61)	49	REF	
English	32,457 (26)	9701 (19)	30	0.45 (0.43, 0.46)	
Mandarin	22,265 (18)	6436 (13)	29	0.43 (0.41, 0.44)	
Vietnamese	2440 (2)	1024 (2)	42	0.76 (0.70, 0.82)	
Spanish	1523 (1)	590 (1)	39	0.66 (0.60, 0.74)	
Other	3082 (2)	1378 (3)	45	0.85 (0.79, 0.91)	
Not documented	340 (0.3)	80 (0.2)	24	0.32 (0.25, 0.42)	
Place of birth					< 0.0001
United States	23,495 (19)	8717 (18)	37	0.85 (0.83, 0.88)	
Outside United States	99,766 (80)	40,777 (82)	41	REF	
Unknown	1434 (1)	273 (1)	19	0.34 (0.30, 0.39)	
Hepatitis B (2)	5683 (8)	2342 (7)	41	0.85 (0.80, 0.90)	< 0.0001
Diabetes mellitus (3)	11,030 (10)	3651 (10)	33	0.92 (0.88, 0.96)	< 0.0001
Current smoker (4)	12,247 (15)	4437 (13)	36	0.82 (0.79, 0.86)	< 0.0001
Insurance status					
Medicaid (5)	71,256 (57)	35,276 (71)	50	REF	< 0.0001
Private	19,916 (16)	6411 (13)	32	0.48 (0.47, 0.50)	
Medicare only	1652 (1)	366 (1)	22	0.29 (0.26, 0.33)	
Other publicly funded	5098 (4)	1417 (3)	28	0.39 (0.37, 0.42)	
Uninsured	26,773 (21)	6297 (13)	24	0.31 (0.30, 0.32)	
Number of years with at least one visit					< 0.0001
1	42,075 (34)	9840 (20)	24	0.24 (0.24, 0.25)	
2	22,727 (18)	8337 (17)	37	0.46 (0.44, 0.48)	
3–4	26,009 (21)	12,703 (26)	49	0.76 (0.73, 0.78)	
5+	33,884 (27)	18,887 (38)	56	REF	

*Percent of total cohort

^a^IQR = inter-quartile range; Age is defined as age during the year of first visit; year of first visit may not align with year of first TB test as some patients were tested prior to their first visit and some patients were subsequently tested

^b^Denominator overall is those with documentation of screening for hepatitis B and age ≥ 18 at on December 31, 2019, N = 73,653; of those, 33,093 were tested for TB infection

^c^Denominator is patients age ≥ 18 at on December 31, 2019, N = 106,326; of those, 37,018 were tested for TB infection

^d^Denominator is those screened for smoking status and age ≥ 18 December 31, 2019, N = 83,457; of those, 33,554 were tested for TB infection

^e^Medicaid includes those with dual Medicaid and Medicare coverage

During the 10-year study period, 49,767 (40%) patients were tested for TB infection with TST or IGRA. Odds of testing were highest in children [0–5 years odds ratio (OR) 4.94, 95% confidence interval (CI) 4.72–5.16; 6–17 years, OR 3.23, 95% CI 3.10–3.36] compared to a reference group of adults aged 18–49 years. In adults, the likelihood of testing decreased the older the age groups (50–64 years OR 0.91, 95% CI 0.88–0.94; 65–79 years OR 0.57, 95% CI 0.54–0.60; ≥ 80 years OR 0.33, 95% CI 0.30–0.37). The presence of comorbidities was associated with a decreased risk of testing (HBV OR 0.85, 95% CI 0.80–0.90 and diabetes mellitus OR 0.92, 95% CI 0.88–0.96). Patients with Medicaid had higher odds of LTBI testing compared to patients with other types of health insurance or no insurance.

The proportion of patients with positive tests, as well as the odds of being prescribed treatment if LTBI testing was positive are presented in Table 2. Overall, 20% of those tested with TST or IGRA at NEMS had a positive test result, with highest positivity in adults aged 50–64 (39%) and 65–79 (39%) years old. Compared to adults aged 18–49 years, patients aged 6–17 years had higher odds of being prescribed treatment (adjusted OR 1.41, 95% CI 1.12–1.79); while adults aged 65 years and older and patients with HBV co-infection had lower odds of being prescribed treatment (65–79 years, aOR 0.72, 95% CI 0.63–0.81; HBV, OR 0.71, 95% CI 0.62–0.82). U.S.-born individuals had lower odds of being prescribed treatment, compared to those born outside the U.S. (aOR 0.83, 95% CI 0.60–1.14).

**Table 2:** Factors associated with being prescribed treatment for latent tuberculosis infection among patients with a positive test, 2010–2019

	Positive result N (%)*	Treatment prescribed N (%)	Unadjusted odds ratio (95% CI)	Adjusted odds ratio** (95% CI)	p-value
Total	10,018 (20)	4658 (46)			
Gender					
Female	5248 (18)	2378 (45)	REF	REF	
Male	4770 (22)	2280 (48)	1.11 (1.02, 1.20)	1.11 (1.02, 1.20)	0.0110
Age^a^	52 [38, 61]	52 [40, 61]			
0–5	82 (1)	42 (51)	1.29 (0.83, 2.00)	1.22 (0.78, 1.91)	0.3952
6–17	313 (4)	169 (54)	1.44 (1.15, 1.82)	1.41 (1.12, 1.79)	0.0043
18–49	4145 (21)	1860 (45)	REF	REF	REF
50–64	3888 (39)	1916 (49)	1.19 (1.09, 1.30)	1.00 (0.91, 1.10)	0.9766
65–79	1453 (39)	632 (44)	0.95 (0.84, 1.07)	0.72 (0.63, 0.81)	< 0.0001
80+	137 (30)	39 (28)	0.49 (0.34, 0.71)	0.37 (0.25, 0.53)	< 0.0001
Race/ethnicity					
American Indian/Alaska Native	3 (6)	0	–	–	
Asian	9371 (21)	4400 (47)	REF		
Black/African American	33 (7)	15 (45)	0.94 (0.47, 1.87)		
Latino	193 (12)	99 (51)	1.19 (0.90, 1.58)		
Native Hawaiian or Other Pacific Islander	30 (11)	3 (10)	0.13 (0.04, 0.41)		
White	111 (7)	37 (33)	0.57 (0.38, 0.84)		
Unknown	277 (18)	104 (38)	0.68 (0.53, 0.87)		
Preferred language					
Cantonese	6868 (22)	3228 (47)	REF	–	
English	1048 (11)	459 (44)	0.88 (0.77, 1.00)		
Mandarin	1363 (21)	575 (42)	0.82 (0.73, 0.93)		
Vietnamese	267 (26)	140 (52)	1.24 (0.97, 1.59)		
Spanish	105 (18)	53 (50)	1.15 (0.78, 1.69)		
Other	354 (26)	199 (56)	1.45 (1.17, 1.80)		
Not documented	13 (16)	4 (31)	0.50 (0.15, 1.63)		
Place of birth					
United States	165 (2)	71 (43)	0.86 (0.63, 1.18)	0.83 (0.60, 1.14)	0.2486
Outside United States	9812 (24)	4579 (47)	REF	REF	REF
Unknown	41 (15)	8 (20)	–	–	
Hepatitis B^b^	879 (38)	359 (41)	0.71 (0.62, 0.82)	–	
No hepatitis B	8166 (27)	4028 (49)	REF		
Diabetes	1379 (38)	642 (47)	1.02 (0.91, 1.14)	–	
No diabetes	8470 (25)	3912 (46)	REF		
Current smoker	1716 (39)	884 (52)	1.13 (1.02, 1.26)	–	
Not a smoker	7052 (24)	3419 (48)	REF		
Insurance status					
Medi-Cal	6853 (19)	3469 (51)	REF	REF	REF
Private	1145 (18)	478 (42)	0.70 (0.62, 0.79)	0.69 (0.61,0.79)	< 0.0001
Medicare only	128 (35)	67 (52)	1.07 (0.76, 1.52)	1.18 (0.82,1.68)	0.3737
Other publicly funded	363 (26)	146 (40)	0.66 (0.53, 0.81)	0.61 (0.49,0.76)	< 0.0001
Uninsured	1529 (24)	498 (33)	0.47 (0.42, 0.53)	0.49 (0.44,0.56)	< 0.0001
Number of years with at least one visit					
1	1621 (16)	561 (35)	0.54 (0.48, 0.60)	0.63 (0.55, 0.71)	< 0.0001
2	1441 (17)	646 (45)	0.82 (0.73, 0.93)	0.91 (0.80, 1.03)	0.1445
3–4	2558 (20)	1263 (49)	0.99 (0.89, 1.09)	1.05 (0.95, 1.16)	0.3725
5+	4398 (23)	2188 (50)	REF	REF	REF

*Positive is defined as a positive result on first IGRA performed (or the first TST, if IGRA was not performed). Denominator for “Positive Result”, for all categories *other than age*, is those “Tested for TB” in Table 1. For an explanation of age categories, see Table Footnote (1)

**Logistic regression model included: Gender, Age, Place of Birth, Insurance status, Number of years with a visit (N = 9977)

^a^Age in this Table is defined as the age at time of first IGRA if performed (or the first TST, if IGRA was not performed). Percentages were calculated with the following denominators that differ from Table 1 due to the different definition of age categorization: 0–5: 8824; 6–17: 7312; 18–49: 19,402; 50–64: 10,054; 65–79: 3718; 80+: 457

^b^Hepatitis B, diabetes and smoking status were only assessed for adult patients, defined as age ≥ 18 at on December 31, 2019

**Fig. 2:**
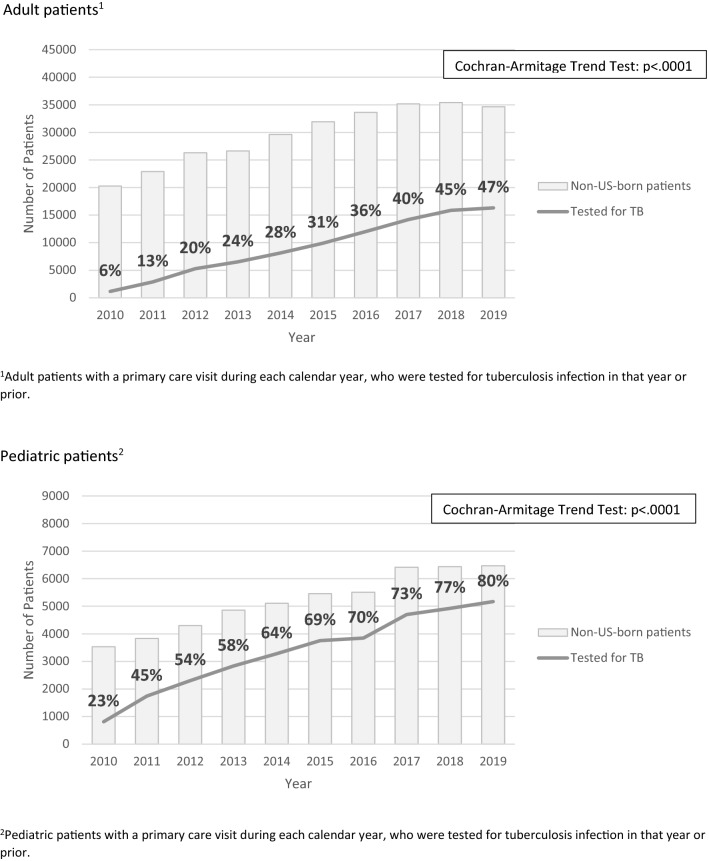
Non-U.S.-born patients seen for a medical visit and tested for tuberculosis over time, 2010–2019

The proportion of non-USB patients seen for a regular or preventive medical visit who were tested for TB infection is presented in Fig. 2. Testing for TB infection among non-USB patients significantly increased over the analysis period, for both adult (6% to 47%, p < 0.001) and pediatric patients (23% to 80%, p < 0.001).

**Fig. 3:**
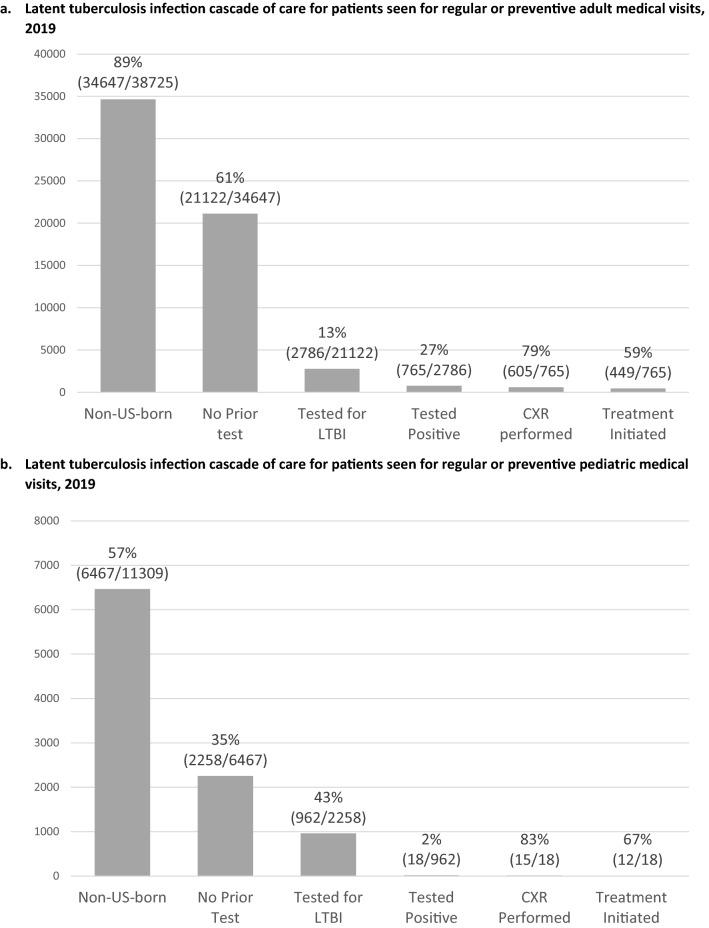
Latent tuberculosis infection cascade of care for adult and pediatric patients seen for regular or preventive medical visits, 2019

A LTBI care cascade for all patients in 2019 is shown in Fig. 3. In 2019, there were 34,647 non-USB adult patients seen for regular or preventative medical visits, of which 21,122 (61%) patients had no prior testing, and of those 2786 (13%) were tested with IGRA or TST in 2019. Of the 765 (27%) who tested positive, 605 (79%) completed a follow-up CXR and 449 (59%) were prescribed LTBI treatment. In contrast, of the 6467 non-USB pediatric patients seen for regular or well-child visits in 2019, only 2258 (35%) had no prior test and of those, 962 (43%) were tested with IGRA or TST in 2019. Test positivity among pediatric patients was lower at 2%, though the proportion prescribed treatment (67%) was higher.

**Fig. 4:**
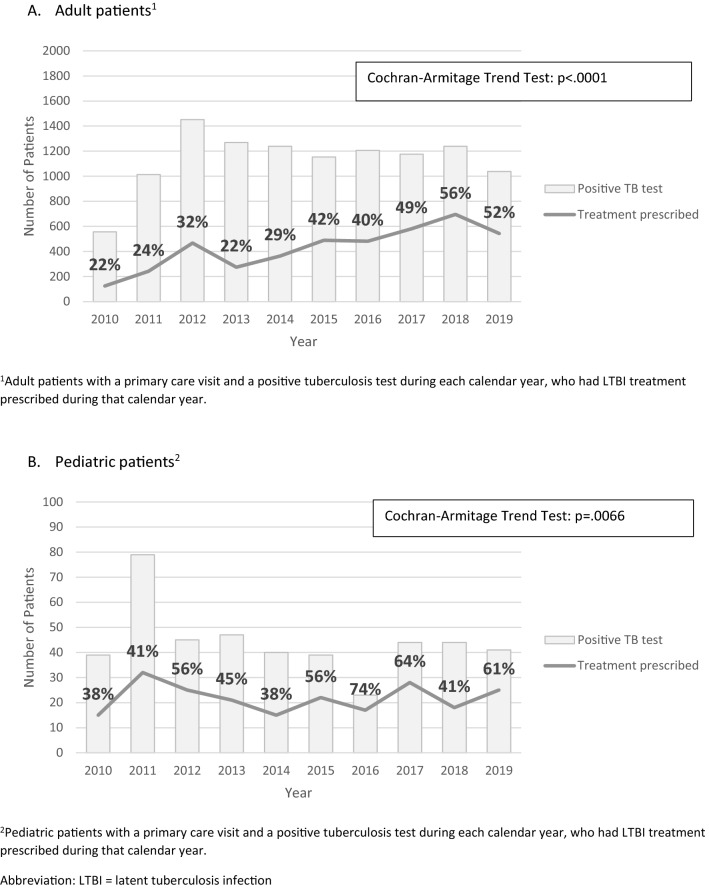
Patients with a positive tuberculosis test with LTBI treatment prescribed over time, 2010–2019

**Fig. 5:**
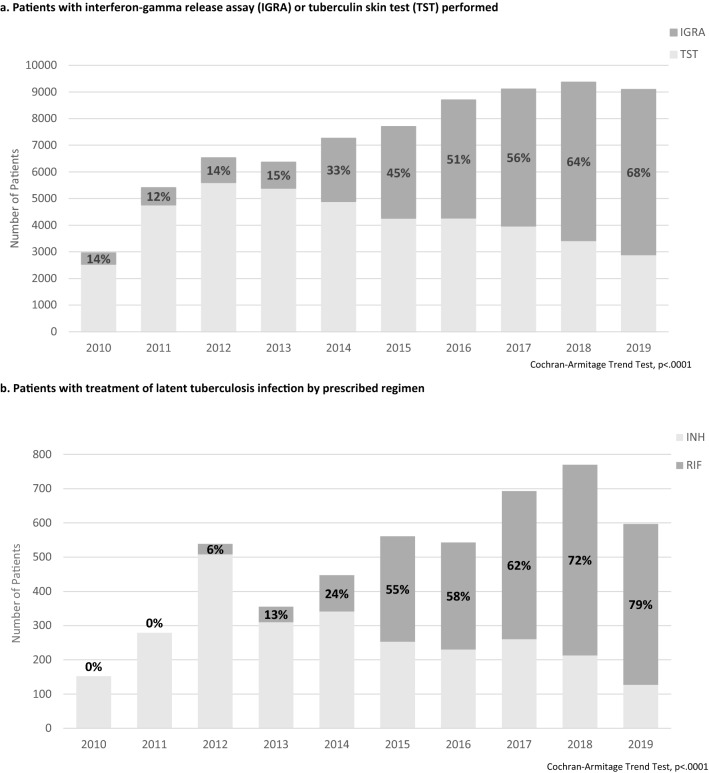
Latent tuberculosis testing and treatment practices over time, 2010–2019

Figure 4 shows the trend in LTBI treatment initiation over the 10-year analysis period. For both adult and pediatric patients with a primary care visit and positive TB test, prescription of LTBI treatment significantly increased during the analysis period (adult patients, 22% in 2010 vs. 52% in 2019, Cochran Armitage p-value < 0.0001; pediatric patients, 38% in 2010 vs. 61% in 2019, Cochran Armitage p-value = 0.0066). Additionally, Fig. 5 demonstrates a statistically significant increase in the proportion of IGRA testing relative to all testing performed during the analysis period (14% in 2010 vs. 68% in 2019, Cochran Armitage p-value < 0.001) (Fig. 5a). Similarly, the proportion of short course rifamycin-based treatment prescribed had a statistically significant increase throughout the study period from 0% in 2011 to 79% in 2019 (p < 0.001) (Fig. 5b).

There was wide variation among providers’ testing of non-USB patients for LTBI and treatment of patients with a positive result (Supplemental Fig. 6), including variation in practice amongst providers at the same clinic site. Of note, there were some providers who tested more patients but had lower rates of prescribing treatment following a positive TB test. Generally, pediatric providers had higher LTBI testing and treatment practices compared to adult medicine providers.

## Discussion

Our study provides a longitudinal 10-year perspective of LTBI testing and treatment trends involving a primarily non-USB Asian population of more than 120,000 patients in a primary care setting. We found a high prevalence of TB infection in our clinic population, particularly among adult patients, with 39% of adults aged 50–79 years testing positive during the study period. Although our analysis did not take place as part of formal implementation research, we observed a significant increase in testing for TB infection and treatment initiation for LTBI during a 10-year period in which NEMS enacted a series of unfunded, clinic-led interventions meant to reduce barriers to LTBI care.

In our study, young age was associated with higher odds of LTBI testing and treatment prescribed. This may be due to school districts requiring all students to complete a TB risk assessment or the fact that pediatric patients see their providers routinely for well-child visits, increasing opportunities for TB testing [19–21]. Our analysis also indicated that older adults had lower odds of being prescribed treatment. This is consistent with meta-analysis data suggesting that older age is a risk factor for not being recommended LTBI treatment [14]. While there is no upper-age cutoff for LTBI testing, providers may perceive older adults to have lower risk for TB disease progression and higher risk of adverse reactions from LTBI medication. Additionally, providers may be uncomfortable managing drug-drug interactions if older patients are taking multiple medications for co-morbidities.

It was troubling to note that although patients diagnosed with HBV and diabetes mellitus had a high prevalence of test positivity, they were less likely to be prescribed LTBI treatment after testing positive for TB infection compared to patients without HBV and diabetes mellitus. This may be due to providers’ perceived risk of liver toxicity from LTBI medications, notably INH, in patients with chronic liver disease, or drug-drug interactions between rifampin and tenofovir alafenamide; provider education should emphasize reduced liver toxicities associated with rifamycin-based regimens and management of drug-drug interactions [13, 22–25]. Future LTBI implementation efforts should include patients with medical co-morbidities including diabetes and hepatitis B; guidance around managing drug-drug interactions and adverse events in these populations are likely needed to increase provider willingness to prescribe LTBI treatment.

In this study, patients with Medicaid health insurance were more likely to receive TB testing and treatment compared to Medicare or privately insured or uninsured patients. While TB testing is a covered benefit under Medicaid in California, Medicare and some private insurers may not cover IGRA under the pulmonary TB screening ICD code. TST is associated with lower levels of LTBI treatment completion compared to IGRA but is the most common TB test found in private insurance claims [26, 27]. Given the wide spectrum of health insurance coverage and healthcare utilization among persons who experience risk of TB in California, the differences in testing and treatment practices based on insurance status merit further study [28].

We are encouraged to observe that over a 10 year analysis period, in a CHC serving a non-USB population with high prevalence of TB infection, that testing and treatment for LTBI increased over time. Our performance in treatment initiation among patients with positive testing in 2019 was similar to that of recently published meta-analysis data from high-income countries; although it is worth noting that the cohort in this meta-analysis included a large proportion of TB contacts, rather than patients seen in primary care [14]. We are also encouraged by a significant increase in utilization of evidence-based practices, including IGRA testing and rifamycin-based regimens over the 10-year period. In keeping with other studies published during this time period, we observed predominant TST use from 2010 to 2015, but from 2016 onwards the majority of TB tests ordered were IGRA [23, 29]. These findings may be attributed to the local health department education, as well as modifying the EHR to include IGRA and rifamycin-based regimens on the commonly ordered tests and medications templates, respectively. Although the cost of IGRA reagents and testing may be cost-prohibitive for some CHCs, use of IGRA has shown to be a cost-effective strategy in non-USB persons [30].

The major limitation of our study is our inability to analyze how interventions directly impacted testing and treatment practices, and which interventions were the most impactful. Since our analysis took place in the context of unfunded and pragmatic clinic-led efforts, rather than in the context of implementation science research, we lack data on the uptake and impact of individual interventions. However, the authors believe that the most impactful interventions were ones that (1) activated patients to engage in their TB care, such as the EHR-automated IGRA result letters which provided test results directly to patients via mail and prompted them to follow-up with their providers for further evaluation, or (2) promoted provider awareness and use of evidence-based LTBI strategies, including annual TB education from the local health department and modification of EHR to enable ordering IGRAs and rifamycin-based therapies. While the CHC implemented several EHR interventions including the annual risk assessment and LTBI treatment info template form, it was difficult to ensure wide and consistent adoption of such tools. Additionally, the provider LTBI progress report, without provider incentives and dedicated time to review the individualized reports, were sometimes overlooked by providers who focused more on Uniform Data System (UDS) measures or organizational goals prioritized by the CHC. Finally, we believe that we saw a change in practice because of the presence of a provider champion, which has been associated with primary care change in other settings as well as commitment to TB prevention as a health equity issue from clinic leadership [31].

A second limitation of our study was the inability to consistently document LTBI treatment completion. Although we were able to capture data on LTBI prescriptions filled by the CHC’s in-house pharmacy, we were unable to track prescriptions filled by outside pharmacies and thus had no consistent nor standardized way to track treatment completion. Further, providers’ documentation of treatment completion varied with some documenting completion in the chronic problem list and others not at all. Prevention of TB cases cannot be achieved unless patients complete LTBI therapy; therefore, retention in care and documentation of treatment completion, which has been proposed in some LTBI care cascade models, should be a focus of future implementation studies [32]. This might include the use of physician extenders or pharmacists as LTBI care coordinators to monitor adherence to and completion of LTBI therapy, as well as discrete EHR field(s) to document LTBI treatment completion.

## New Contribution to the Literature

The high prevalence of TB infection in this study illustrates the critical role CHCs serving non-USB populations have in TB prevention. This urban CHC introduced a series of internally driven EHR and provider-centered QI interventions without external funding and saw significant improvements and sustained practice changes in LTBI testing and treatment over a 10-year period. Furthermore, this study offers insight on LTBI care cascades at a primary care-led setting using a large, eligible patient population (n = 124,695) rather than a patient population starting at the time of LTBI testing. Although national progress towards TB elimination in the U.S. may require larger systemic changes, such as making TB screening a UDS or Healthcare Effectiveness Data and Information Set (HEDIS) measure or developing a LTBI quality indicator, the experience of this health system provides a hopeful model for CHCs to prevent TB cases among their patient populations.

## Supplementary Information

Below is the link to the electronic supplementary material.

Supplementary file1 (PDF 295 KB)

Supplementary file2 (PDF 97 KB)
