# Supplementary material for: Can Primary Care Drive Tuberculosis Elimination? Increasing Latent Tuberculosis Infection Testing and Treatment Initiation at a Community Health Center with a Large Non-U.S.-born Population
Source: J Immigr Minor Health. 2023 Jan 18;25(4):803–15. doi: 10.1007/s10903-022-01438-1 (PMC9847435; doi:10.1007/s10903-022-01438-1)
Supplement: Supplementary file 1 — Supplementary file1 (PDF 295 KB) [file 10903_2022_1438_MOESM1_ESM.pdf]

Supplement Figure

Figure 6a. Proportion of non-US-born patients tested and treated for LTBI, by adult provider* and clinic site

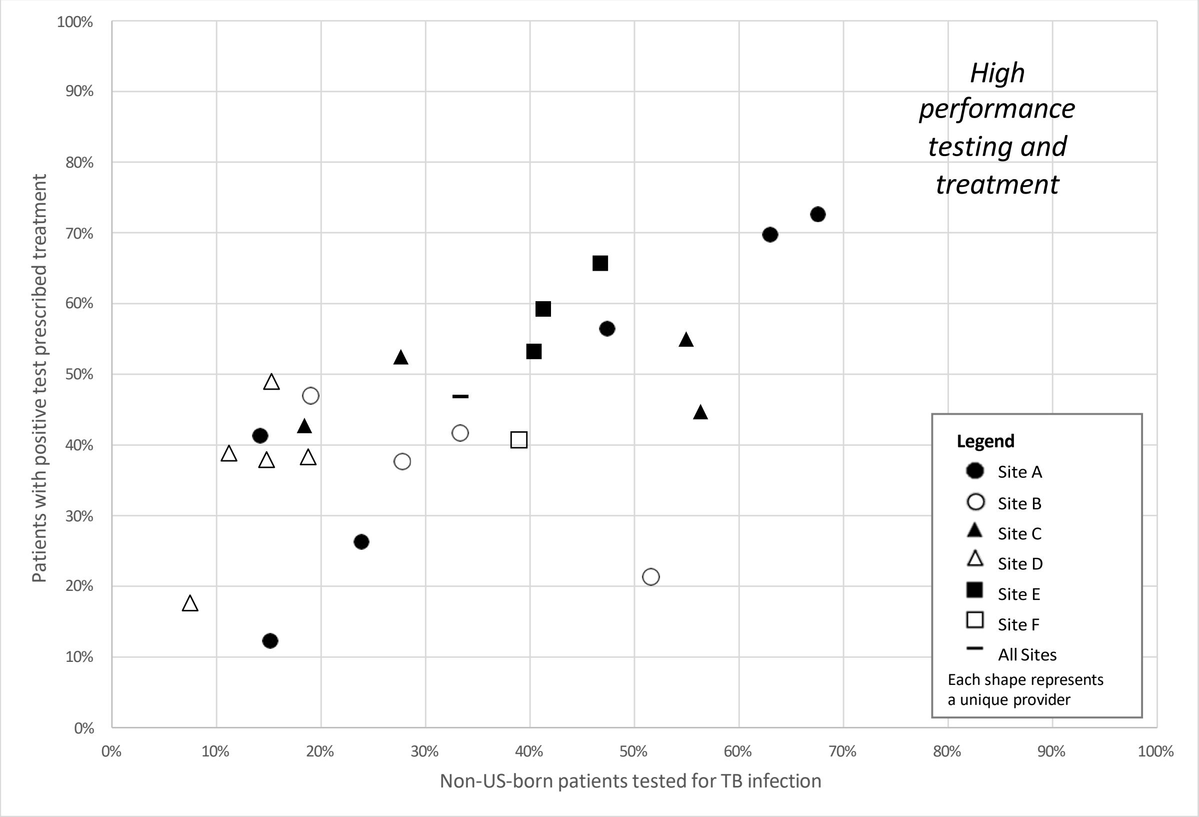

*Providers included are those that were assigned as the primary care provider for >1800 patients in the cohort at the time of data extraction (N=23).

Figure 6b. Proportion of non-US-born patients tested and treated for LTBI, by pediatric provider* and clinic site.

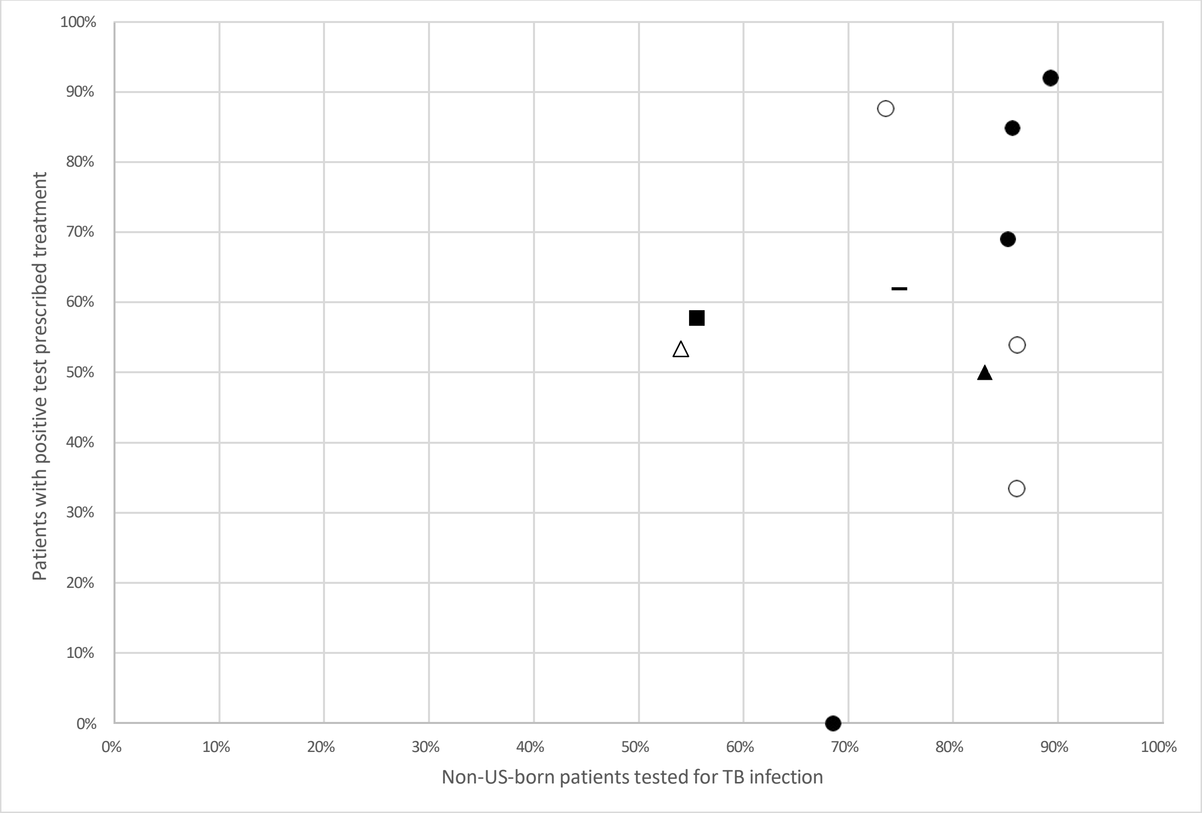

*Providers included are those that were assigned as the primary care provider for >1300 patients in the cohort at the time of data extraction (N=10).
