# Supplementary material for: Can Primary Care Drive Tuberculosis Elimination? Increasing Latent Tuberculosis Infection Testing and Treatment Initiation at a Community Health Center with a Large Non-U.S.-born Population
Source: J Immigr Minor Health. 2023 Jan 18;25(4):803–15. doi: 10.1007/s10903-022-01438-1 (PMC9847435; doi:10.1007/s10903-022-01438-1)
Supplement: Supplementary file 2 — Supplementary file2 (PDF 97 KB) [file 10903_2022_1438_MOESM2_ESM.pdf]

Supplemental Table 1

LTBI Care Cascade Step	Numerator	Denominator	Comment
**Eligible population: non-US-born**	Non-US-born	All patients (Non-US-born + US-born)	We did not exclude low risk non-US regions due to incomplete place of birth data and majority non-US born from Asia
**Cascade eligible population: No prior TB test**	No prior TB test	Non-US-born	Although some cascades exclude this step, we decided to include a step for no prior TB test, to focus on highest need population for testing
**Tested for LTBI (Test completed)**	Test completed	No prior TB test	Did not include tests ordered (but not completed) and did not exclude invalid/indeterminate tests here
**Tested positive**	Tested positive	Test completed	IGRA or TST
**CXR performed**	CXR performed	Tested positive	CXR only (not chest CT or other imaging); included CXRs performed 6 months prior to 1 year following positive test date.
**Treatment initiated**	Treatment prescribed	Tested positive	Unable to provide consistent treatment completion data so not analyzed
**Treatment Completed**	Treatment completed	Treatment initiated	As above
